# Evaluating the effectiveness of a single-day simulation-based program in psychiatry for medical students: a controlled study

**DOI:** 10.1186/s12909-021-02708-6

**Published:** 2021-06-16

**Authors:** Nadia Younes, Anne Laure Delaunay, M. Roger, Pierre Serra, France Hirot, Frédéric Urbain, Nathalie Godart, Mario Speranza, Christine Passerieux, Paul Roux

**Affiliations:** 1 Université Paris Saclay, Université Versailles Saint Quentin, CESP, Team DevPsy, 94807 Villejuif, France; 2 Centre Hospitalier Versailles, Service Hospitalo-Universitaire de Psychiatrie de l’Adulte et d’Addictologie, 177 Rue de Versailles, 78157 Le Chesnay, Cedex France; 3 Center for medical simulation of UVSQ, Université de Versailles Saint Quentin en Yvelines UFR des Sciences de la Santé Simone Veil, Montigny le Bretonneux, France; 4 Centre Hospitalier Versailles, Service Hospitalo-Universitaire de Psychiatre de l’Enfant et de l’Adolescent, F-78157 Le Chesnay, France; 5 Fondation Santé des Etudiants de France (FSEF), Paris, France; 6 Département de médecine générale, UFR des sciences de la santé Simone Veil, Université Versailles-Saint-Quentin-en-Yvelines, Paris, France

**Keywords:** Simulation, Simulated patient, Psychiatry, Medical students, Psychometrics, Confidence, Clinical skills, Internal consistency, Factor analysis, Test-retest reliability

## Abstract

**Background:**

Training in psychiatry requires specific knowledge, attitudes, and skills that are obtainable by simulation, of which the use is only recent and still needs further development. Evidence is accumulating on its effectiveness but requires further validation for medical students. We aimed to evaluate the effectiveness of a single-day optional teaching program in psychiatry by simulation for medical students and validate a scale measuring Confidence in Psychiatric Clinical Skills (CPCQ), as part of the assessment.

**Methods:**

This was a controlled study in a French University that compared (using paired-sample Student t-tests) knowledge and attitudes (university grades and CPCQ scores) before, just after teaching with simulated patients, and 2 months later. Satisfaction with the program (including the quality of the debriefing) was also investigated. The CPCQ scale was validated by assessing the factor structure, internal consistency, and test-retest reliability. Finally, a comparison was run with a control group who received the usual psychiatric instruction using covariance analyses.

**Results:**

Twenty-four medical students were included in the simulation group and 76 in the control group. Just after the simulation, knowledge and attitudes increased significantly in the simulation group. Satisfaction with the training and debriefing was very high. The CPCQ scale showed good psychometric properties: a single-factor structure, acceptable internal consistency (α = 0.73 [0.65–0.85]), and good test-retest reliability (ICC = 0.71 [0.35–0.88]). Two months after the simulation, knowledge and attitudes were significantly higher in the simulation group than the control group, despite a lack of difference in knowledge before the simulation.

**Conclusions:**

Adding a simulation program in psychiatry to the usual teaching improved the knowledge and confidence of medical students. The CPCQ scale could be used for the evaluation of educational programs.

**Supplementary Information:**

The online version contains supplementary material available at 10.1186/s12909-021-02708-6.

## Background

In Medical Education, the use of the pedagogical method of simulation has increased greatly since the introduction of” Harvey”, the cardiology patient simulator, in the 1970s, followed by other experiments in surgery, pediatrics, obstetrics, and anesthesia, and now concerns all medical fields [37]. It includes technology-enhanced simulation (virtual reality simulators, mannequin-based simulation, or computer simulation with virtual patients [19]) as well as standardized patients (SPs), notably in psychiatry [2].

Simulation promotes learning through the experience, which facilitates knowledge production. According to Kolb’s experiential learning theory, “learning is the process whereby knowledge is created through the transformation of experience”. It implies four stages starting with concrete experience (doing/having an experience/feeling), followed by reflective observation (watching/reflecting on the experience) which leads to the formation of Abstract Conceptualisation and generalizations (thinking, concluding and making sense of what has happened). The last stage is active experimentation (considering how to put what has been learnt into practice) [47]. The debriefing, that follows the simulated or real experience, consists of a facilitated conversation in which participants analyze their actions, thought process, and emotional states during three stages. It is a major component which emphasis reflective observations, conceptualization, and active experimentation [1, 13, 48, 68]. Two methods are well-established: the Debriefing Assessment for Simulation in Healthcare (DASH) [13] and the Objective Structured Assessment Debriefing (OSAD) [9]. 

In psychiatry, simulation is a relatively new field [53]. Despite a dramatic evolution over the last couple of years, it continues to have show ongoing development and changes [2, 50]. Training in psychiatry requires particularly specific knowledge, attitudes, and skills that cannot simply be learned theoretically without experiential learning. Simulation provides an excellent opportunity to develop specific communication [55], psychotherapeutic, clinical, technical [46, 64], and teamwork skills [10] to assess and manage various psychiatric disorders. Simulation is also efficient to make practitioners from other fields than psychiatry to acquire skills in mental health. For instance, mental health simulation programs have been developed for general practitioners [51], emergency physicians [17], paediatricians [18]. A recent meta-analysis on simulation training in psychiatry for medical students, post-graduate trainees, and qualified doctors reported a threefold increase in research over the past 10 years [62]. Several universities have introduced such training as compulsory [39] because medical students are demanding it for several reasons. All students, and not only those who want to become psychiatrists, may benefit from such learning because psychiatry is de facto practiced by many physicians, starting with GPs [65]. A significant proportion of medical students never participate in a clerkship in psychiatry due to the limited number of places and even for those who do, training may be insufficient [3, 50]. Simulation could reduce fear and stigmatization. It also avoids the potential inconvenience of inexperienced students interacting with vulnerable individuals and, above all, exposing them to large groups of students [12, 24].

The standard reference for the assessment of a learning intervention is Kirkpatrick’s Training Evaluation Model [45], which measures the impact on five levels: 1) reaction effect: satisfaction/dissatisfaction of participants; 2) learning effect: participants improve their knowledge; 3) behavioral effect: changes in attitudes, skills, or learners’ confidence in their own psychiatric clinical skills and anticipatory anxiety [43, 61, 70]; 4) patient results (i.e. if the intervention improves diagnosis or management of the disorders) to approach operational effectiveness and to reach patient-reported outcomes, which are essential in the evaluation of an intervention [56]; and 5) return of investment. The meta-analysis showed the global effectiveness of simulation training in psychiatry on attitudes, skills, knowledge, and satisfaction [62]. We analyzed the results for medical students (48 surveys, 16 RCTs, and 32 controlled studies, 10 with follow-up). Most interventions (*N* = 44) were one session, 4 were repeated. A positive impact on satisfaction was reported for level 1 in seven studies. However, structured investigations of medical students’ satisfaction with the debriefing in psychiatry simulation-based teaching are scarce in the literature. Studies investigating medical students’ satisfaction with simulation mainly used non-validated tools [8, 28, 32, 58]. The present study thus addresses a significant literature gap about debriefing satisfaction in medical students learning psychiatry with simulation, by evaluating debriefing satisfaction with DASH.

For level 2, an improvement in student’s objective knowledge after simulation relative to other pedagogical methods was reported in 14 studies (and none in 6). However, this improvement was usually assessed immediately after the intervention. Only four studies evaluated this outcome with a gap between 1 and 4 months. Still, they did not strictly focus on general psychiatry, as two of them investigated alcohol abuse [7, 41], the other one opioid abuse [54], and the last one geriatric medicine [30]. The present study addresses this literature gap by investigating the long-term effectiveness of a simulation-based education across the whole national psychiatry program for medical students. 

For level 3, 28 studies assessed attitudes such as empathy and skills, and 10 reported an improvement in self-confidence [26, 29, 61] without an assessment over time.

The review reported any study evaluating level 4. However, a study conducted among 3rd year medical students in Israel showed the effectiveness of a single-day simulated-based training in improving communication skills with real patients [8].

We developed and evaluated an intervention for medical students with three features: 1) a single-day program, 2) coordination between teachers of psychiatry for adults, children, and adolescents and those of general medicine, and 3) presentation of common situations encountered in daily practice in primary care or in the emergency room. We also developed a scale to measure the learners’ confidence in their psychiatric clinical skills. Examining one’s own practices is a fundamental dimension to characterize skills and clinical performance ([49, 73]. No tool was suitable for our study. Confidence scales from nursing education with excellent psychometric properties [27, 31, 36] were not adapted for medical students. General self-assessment scales of psychiatric competence did not study their psychometric properties [6, 72]. Finally, confidence scales with satisfactory internal consistency but specific to certain clinical situations (such as suicidal risk [52, 57, 63] and depression [61] could not be used for our clinical situations.

The present study thus had two objectives: 

1) to evaluate the effectiveness of the single-day simulation program for medical students in terms of satisfaction (including satisfaction with the debriefing), knowledge (after the intervention and with a long follow-up: exam grades 2 months later, and attitudes (self-confidence in clinical skills and changes in professional practices) and 

2) to explore the psychometric properties of a new self-report questionnaire on confidence in one’s clinical skills in psychiatry.

## Methods

### Design

**Fig. 1:**
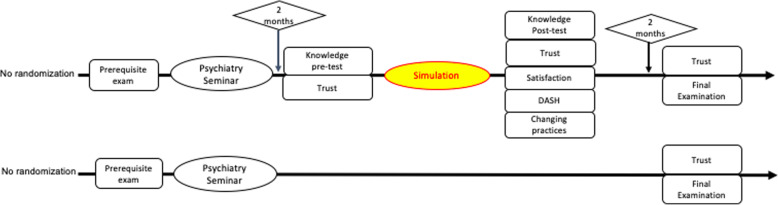
Design of the study

The study had a mixed design, with comparisons of before and after the intervention and between the intervention and control groups (Fig. 1).

### Population

The population was recruited during the 2019–2020 academic year among the 131 fifth-year undergraduate students at the University of Versailles Saint-Quentin-en-Yvelines-Paris Saclay (the year of compulsory psychiatric training).

### Intervention

The intervention consisted of single day (8 h) of teaching of psychiatry by simulation with a simulated patient, according to the definition proposed by Adamo of a “medical encounter conducted for purely educational purposes” [4]. The scenarios, decided within a group of 10 hospital-university teachers (from university fellow to professor in general medicine, child and adolescent psychiatry, or adult psychiatry), had to be: 1) addressed in the curriculum of the official national program [5], 2) a pathology frequently encountered in general practice, and 3) realistically performed by a team of psychiatry teachers without specific background in dramatic arts (eliminating the scenario of schizophrenia, which is challenging to play [62]). A complete example of a scenario is presented in Supplementary Material. The scenario had the following general structure:

- Information about the learners (their expected level of formation, the prerequisite, and their role in the simulation)
- Information about the trainers (their level of formation and their function in the simulation: simulated patient, briefing, re-briefing, facilitator, debriefing …)
- Information about how the room should be prepared
- Pedagogical objectives (medical & technical / non-technical) with references according to the french national program for advanced medical student
- The critical points of the briefing and debriefing
- A summary of the clinical situation for the trainers
- The content of the pre-briefing, with the description of the beginning of the situation that has to be communicated to the learner
- A precise description of the role of the simulated patient (biography, social context, medical history, personality)
- The description of the expected progress of the simulation (how the simulated patient should behave emotionally, what she/he should say, when she/he should move from one stage to another according to the learner behaviour

As in several previous investigations, simulated patients were portrayed by mental health professionals [34, 58, 59, 61, 71, 74]. Simulated patients were trained to portray the simulated patient by two academic teachers (PR & MR), themselves trained as health simulation trainer in a short (one-week) university course. Moreover, 6 academic teachers in the team had a former teaching psychiatry experience with simulation. The choice to use a teacher rather than a professional actor was based on a concern for realism, as psychiatry teachers should have a better knowledge of symptoms of psychiatric disorders than a professional actor. Some authors have indeed suggested that actors sometimes tend to overplay their role or go out of their script [25], or even caricature mental disorders [21]. Besides, the additional costs of actors could not be supported in the present study, with an estimation of up to £250 per scenario for initial training and £75 for each new simulation session [21].

The four scenarios presented a drug suicide attempt in the context of borderline personality disorder and alcohol addiction assessed in the emergency room by a psychiatrist (Supplementary Information) and bereavement associated with post-traumatic stress disorder, hypomania, and a refusal to go to school by a 14-year-old adolescent assessed in a general practice setting.

Each simulation session included a briefing (10 min), the simulation (10–20 min), a structured debriefing (45 min), and a theoretical synthesis slide presentation (20 min). We used the Karlsen’s RUST (Reaction – Understanding – Summarise – Take home message) model of debriefing [44]. During the understanding phase, we used the “debriefing with good judgment” [67]. This approach relies first on the detection of performance deficits in students. The teacher then tries to uncover the underlying knowledge, assumptions, and feelings that drove students’ actions. Finally, the teacher gives feedback about the performance deficits and tries to bring the underlying student’s intentions and cognitive frames to light using a conversational technique pairing advocacy and inquiry.

Learners were divided into groups of eight (2 actively participating, 6 watching the live video broadcast in an adjacent room). The play was based on voluntary participation; students were asked to play at least in one scenario, and it was the condition to validate the optional teaching unit. Three teachers were involved, one playing the patient’s role, one as a potential facilitator, and one staying with the learners. No randomisation was used to assign the intervention or control group status. Students in the intervention group accepted an optional teaching unit on the condition that they actively participated in one scenario. The control group received the same usual psychiatric instruction as the simulation group in the form of a compulsory two-day interactive seminar with the flipped classroom technique [38]. All included participants were provided with the pedagogical written content of the simulation sessions to ensure that any differences between the groups were related to the simulation teaching technique itself.

### Measures

#### Knowledge

Theoretical knowledge was measured using multiple choice questions (MCQs) three times: for all students, 2 months before the simulation teaching (45 questions before the compulsory psychiatry seminar) and 2 months after (50 MCQs during the psychiatry examination, covering the entire national program of psychiatry, that is a challenging test) and for the simulation group, before and after the teaching (28 same questions but asked in a different order). All scores were scaled from 0 to 20.

#### Attitudes (Supplementary Information 1)

Confidence was assessed by the specifically created Confidence in Psychiatric Clinical skills Questionnaire (CPCQ): 12 items, rated on a four-level Likert scale, explored confidence in theoretical knowledge, clinical skills (clinical reasoning and psychiatric interviewing), communication and interpersonal skills (with the patient, the patient’s proxies, and other professionals), and the management of psychiatric disorders. The individual mean score was used in the analyses. The change in professional practice was evaluated with one question.

#### Satisfaction (Supplementary Information 2 and 3)

General satisfaction was rated out of 10. A 10-item questionnaire, rated on a four-level Likert scale, explored various aspects of satisfaction, such as the preference for simulation over another pedagogical modality, the perceived realism of the situation, the importance of being actively involved, etc. In addition, learners who underwent a clerkship in psychiatry were asked to compare it to the simulation. Questions about the scenarios and free comments were collected.

Satisfaction with the briefing and debriefing was assessed using the student version of the DASH [69]. This scale, with excellent internal consistency (0.82–0.95) [14, 23, 66], explores the climate, structure of the debriefing, ability to engage in exchange, and strengths and areas for improvement. The mean across all items (6 overall assessments, 23 behavioral assessments) was used.

### Statistical analysis

#### Pre/post-simulation comparisons

The average CPCQ and knowledge test scores just before and after simulation were compared using paired sample Student t-tests. Satisfaction was measured post-simulation.

#### Psychometric characteristics of the CPCQ scale

Construct validity was explored by exploratory factor analysis using oblim rotation and maximum likelihood factorization as the factorization method. Two criteria were used to determine the number of significant factors: first, Catell’s scree test, i.e. factors present to the left of the eigenvalue curve deflection [22], and second, Kaiser’s criteria, i.e. factors for which the eigenvalue is > 1[42]. The internal consistency of each identified factor was evaluated using Cronbach’s α coefficient [20], with an acceptability threshold set to 0.7 [11]. These analyses were carried out on the largest sample for the same time of measurement (final exam) and by bringing the two groups together.

Test-retest reliability was assessed by the intra-class correlation coefficient (ICC), calculated using a mixed model with a random double effect. It was defined as poor for an ICC <  0.4, acceptable between 0.4 and 0.59, good between 0.6 and 0.74, and excellent between 0.75 and 1 [15]. The two times of measurement chosen to calculate it were those for which the least possible change was expected, i.e., just after the simulation and 2 months later.

#### Comparisons between simulation and control groups

First, age and participation in a clerkship in psychiatry (a potential confounding factor for confidence [57]) were compared between the two groups using chi^2^ tests and scores on the pre-requisite exam using Student’s t-test. Analyses of covariance (ANCOVA) was then carried out with the mean CPCQ score and the psychiatry final exam score as dependent variables, the group as the independent variable, and the covariates that differed significantly between the two groups (clerkship in psychiatry).

#### Number of required subjects

The number of required subjects was calculated for knowledge (score out of 20 on the usual psychiatric examination). According to the results of the previous year, the average score was 13.3, with a standard deviation of 1.9. To show a mean difference of 2 points with an alpha risk of 5% and a statistical power of 90% required at least 19 subjects per group.

### Ethics statement

The research was authorised on 20/12/2019 by the Ethics Committee of the University of Paris-Saclay (CER-Paris-Saclay-2019-061). All participants signed written and informed consent.

## Results 

### Participants

The study was proposed to all 131 medical students in their fifth-year at the University of Versailles Saint-Quentin-en-Yvelines-Paris Saclay through an announcement by the educational department. Among these students, 100 accepted to participate (): *N* = 24 received the intervention, and *N* = 107 did not. The first 24 included students who accepted to participate in the intervention were assigned to the intervention arm. As the teaching was optional, it was not possible to randomise participants between two arms, as the inclusion within the intervention arm should be entirely based on student preferences. The simulation and control groups did not differ in terms of either the sex ratio (X^2^ = 0, *p* = 0.93) or initial knowledge (t (97) = 1.2, *p* = 0.24). There were more students with a clerkship in psychiatry in the intervention group (X^2^ = 3.7, *p* = 0.056) (Table 1).

**Table 1:** Comparison of the Simulation group (*N* = 24) and Control group (*N* = 7 6) among the 131 fifth-year medical students at the University of Versailles Saint-Quentin-En-Yvelines for characteristics, knowledge, attitudes (confidence, change in professional practice) and satisfaction in the simulation group

		Simulation Group (S) ***N*** = 24			p (pre-post)	Control Group (C) ***N*** = 76			p (S-C)
**Group characteristics**	Gender (N, % males)	*N* = 24 (41.7%)				*N* = 74 (37.8%)			ns
	Clerkship in psychiatry (%)	*N* = 2 4 (20.8%)				*N* = 76 (5.2%)			**0.006**
(Continuous variables)		N	M	SD		N	M	SD	
**Knowledge** [0–20]	Grade at initial university check	24	15.6	1.2		75	15.2	1.5	ns
	Pre-simulation	24	16.3	1	**0.**01				
	Post-simulation	24	16.8	0.6					
	University Grade (2 months after)	24	13.7	1.4		76	12.8	1.4	**0.016**
**Confidence CPCQ**[1–4]	Pre-simulation	24	2.2	0.3	**< 0.001**				
	Post-simulation	24	2.7	0.2					
	2 months after simulation	17	2.7	0.2		75	2.4	0.3	**0.003**
**Change professional Practices [1-4]**		24	3.4	0.5					
**Satisfaction**	General [0–10]	24	9.3	0.6					
	Satisfaction Questionnaire [1–4]	24	3.5	0.2					
	Total DASH [1–7]	24	6.5	0.4					

*M* Mean, *SD* Standard deviation, *CPCQ* Confidence in Psychiatric Clinical skills Questionnaire

### Knowledge (Table 1)

Theoretical knowledge (t (23) = 2.6, *p* = 0.01) improved after teaching. The ANCOVA showed better theoretical knowledge on the psychiatry exam (F (1.96) = 6, *p* = 0.016) in the simulation group than in the control group.

### Attitudes (Table 1)

Confidence measured with the CPCQ scale improved after teaching (t (23) = 8.2, *p* <  0.001). Learners shifted from an average low confidence level (2.2 ± SD 0.3) before instruction to an average high confidence level (2.7 ± 0.2) afterwards. This gain was maintained for 2 months, insofar as the reassessment of confidence with the CPCQ scale was not significantly different between immediately after the simulation and 2 months later (t (15) = 0.7, *p* = 0.52). The ANCOVA showed higher confidence on the CPCQ scale (F (1.89) = 6, *p* = 0.003) for the simulation group than the control group.

### Satisfaction (Table 1)

The overall satisfaction score was excellent (9.3 + −SD 0.6). The average scores on the satisfaction questionnaire (3.5 ± SD 0.2) showed that learners were satisfied to very satisfied with the teaching. The lowest score was obtained on the question about optional or compulsory teaching “(2.7 ± SD 0.8), suggesting a neutral position for the group of learners. The highest score was obtained for the questions on the preference of courses rather than the simulation (3.9 ± SD 0.3) and on the simulations’ realism (3.8 ± SD 0.4). All learners who had a clerkship in psychiatry felt that the simulation was more (4/5) or much more (1/5) informative than the clerkship.

The level of difficulty was found to be appropriate on average (3.1 ± SD 0.2). The scenarios were judged to be informative or very informative (3.5 ± SD 0.4).

Free comments were positive and suggested improvement areas (summarising an ideal psychiatric interview, furthering theoretical reminders, including other pathologies, such as eating disorders and schizophrenia).

The average total DASH score showed the briefing and debriefing of the simulation sessions to be rated as very good (6.5 ± SD 0.4). Behavioural scores suggested a good sense of security for the learners.

### Psychometric characteristics of the CPCQ scale

#### Factor structure

**Fig. 2:**
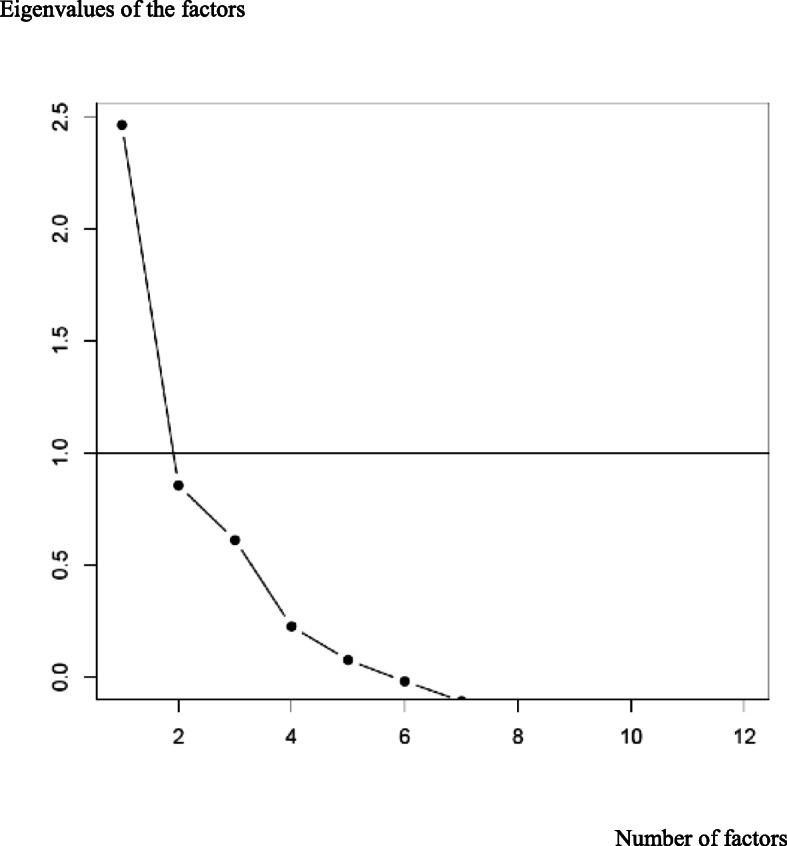
Slump Diagram of the Confidence in Psychiatric Clinical Skills scale

A scree diagram (Fig. 2) showed a single-factor structure of the CPCQ scale according to the Catell criterion, as the deflection of the curve occurred for two factors. The first factor was the only one with an eigenvalue > 1 (Kaiser criterion) and accounted for 20.5% of the variance.

#### Internal consistency

With a Cronbach’s alpha coefficient of 0.73 [0.65–0.85], the internal consistency was satisfactory.

#### Test-retest reliability

The ICC, equal to 0.71 [0.35–0.88], suggests good test-retest reliability.

## Discussion

We evaluated the effectiveness of a single-day simulation program with simulated for medical students as a complement to usual teaching. There was an improvement after simulation for knowledge and attitudes. Satisfaction, including that concerning the debriefing, was high.

The effectiveness in improving knowledge of our simulation training in psychiatry is coherent with previous results with medical students [62]. We confirmed the maintained positive impact at 2 months, showing for the first time an improvement of academic performance across the whole national program of psychiatry, i.e. a challenging test, 2 months later. Simulation in psychiatry, an essential addition to other pedagogical tools, allows sustainable acquisition of knowledge that cannot be simply learned theoretically and memorized without experiential learning [16]. All participants who had a clerkship in psychiatry and the simulation found the latter to be more informative, which questions the clerkship’s pedagogical benefit. Simulation with a simulated patient provides an opportunity for real-time feedback and reflection on performance, which is rarely the case in interactions between medical students and people with psychiatric disorders [21]. The improvement in attitudes is also consistent with previous studies on medical students [62]. This is the first study to report that self-confidence improvement is maintained 2 months after receiving the training. Among attitudes, self-confidence is that which has been the most explored relative to empathy or stigmatisation [62], and has been associated with better skills, for example, in assessing suicide risk [49]. It is important to notice that the improvement in knowledge and attitude occurred despite the simulated patients were played by teachers and not professional actors. Our results thus suggest that using academic teacher could be an efficient alternative in settings were actors are unavailable. Simulation is popular among students. Our study confirmed a very high level of satisfaction with the content of the teaching and its usefulness for practice. The average DASH score, well above the usual acceptability threshold of four [14], suggests effective briefings and debriefings in a safe educational framework. Despite the lack of teachers trained to act, the simulations appeared to be realistic to the learners. Future studies could use validated scales, such as the *Maastricht Assessment of Simulated Patients* [75], to assess the quality of simulated patient roleplay reliably. Compared to evaluated simulation programs, our program has the particular form of a single-day training while the others are mostly shorter (one session) or longer (repeated). Only one other program with a similar format to ours in its general approach to different psychiatric disorders reported a positive impact upon communication and psychiatric skills for medical students [8]. The CPCQ scale of the confidence of medical students’ clinical skills in psychiatry showed satisfactory psychometric properties (acceptable internal consistency, good test-retest reliability, and a unifactorial structure) and it proved to be an easy and rapid evaluation tool. It is an important addition to tools for which the psychometric properties are not known [40]. Given the small sample size for measuring test-retest reliability, the confidence interval obtained was large and the results should be replicated on a larger sample.

Our study had several limitations, despite a relatively high median quality, as assessed by the MERSQI (Medical Education Research Study Quality Instrument), i.e. 12 (Supplementary Information 4) vs 10.8 for studies reported in the meta-analysis [62]. As in most previous studies [62], the main limitations were the absence of random assignment and a control condition that was teaching as usual, not a control pedagogical intervention with the same duration as simulation. It was not possible to randomise since this teaching was optional and therefore based on student preferences. The higher proportion of learners with a clerkship in psychiatry in the simulation group may suggest a selection bias toward individuals with a high level of interest and motivation for the discipline. Second, some measures were missing: prior exposure to simulation experiments for both groups and a measure of pre-intervention confidence for the control group (the simulation group may have had a higher level of confidence than the control group prior to the intervention, in connection with participation in a clerkship in psychiatry). Third, the generalizability of our results is limited by the small sample size and a single teaching site. Fourth, our study lacked a hetero-evaluation of psychiatric clinical skills. We did not find a validated scale to assess skill in psychiatry, despite the efforts of certain authors to develop objective measures of the efficiency of a psychiatric interview [59], and an assessment by teachers was not possible, as the students participated in the simulation only once. Moreover, we did not explore level 4 of Kirkpatrick’s model for simulation, i.e. the outcome on management and individuals with a mental disorder. This would be important to allow wider dissemination of this pedagogical technique in the mental health field. The fact that teachers played the simulated patient has its limitations. It would have affected the training’s realism due to a lack of specific dramatic arts background in these non-professional actors. This interpretation is not supported by the fact that students reported they found the situational scenarios very realistic (3.8/4 + − 0.4), suggesting that teachers’ acting was satisfactory. A social desirability bias might have explained the high level of satisfaction reported in this study, as the teachers played the simulated patients’ roles. However, all questionnaires were anonymously filled, which should have limited this bias. Moreover, if this bias might have led to a global tendency to overestimated satisfaction, it would not explain why the item focusing on the realism of the simulation reached the second highest score of satisfaction. If there had been a realism issue, this item would have been scored lower than the other ones. One might have also argued that the present study’s simulation format was more a structured roleplay [35] than a simulated interview. The main difference between a simulated interview and a roleplay would be the predetermination of the role and the scenario [33]. In a roleplay, there is symmetry between the two players who can perform either the patient’s or doctor’s role. The clinical situation is partly unpredictable, with possible improvisation and weak determination of the fictional role. It is based on the personal and professional experience of each person. In contrast, a simulated patient strongly relies on a structured scenario, with prewritten dialogues and a precise emotional, biographical, and personality portrayal. Within a simulated interview, it is impossible to switch the role, as there is a strong asymmetry between the simulated patient’s and doctor’s background. Our study used detailed scenarios for the four simulated patients, with prewritten dialogues, fixed progress, and facilitators in order to make the scenario move forward in case of a stalled situation. We choose the simulated patient format rather than the roleplay format because our experience with roleplaying was a low medical student’s satisfaction. This experience was confirmed by the responses to the satisfaction question “Would you have preferred roleplaying (with some students playing the role of patients) rather than simulation teaching to complement your psychiatric training?” which were between “disagree” and “strongly disagree” (3.5/4 + − 0.5).

Future studies should compare the pedagogical efficiency of simulation programmes in psychiatry using simulated patients played by health professionals against simulated patients played by professional actors. They should also focus on level 4 of Kirkpatrick, for instance, by investigating the impact of simulation on students’ psychiatric competencies during interviews with the real patients they encounter during their clerkship in psychiatry. In a future research direction, it would be interesting to compare the pedagogical efficiency and the satisfaction in terms of secure feelings between simulation involving one and two simultaneous active students like in this study. In this study, we did not use video recording for debriefing: it would be important to elaborate on how this tool that avoids cognitive bias in remembering could be integrated into a debriefing with medical students without hampering the debriefing fluidity.

## Conclusion

Our study shows the effectiveness in terms of knowledge gained, attitudes, and satisfaction of a single-day program of teaching psychiatry through simulated-based simulation as a complement to usual teaching for fifth-year medical students in France. The teaching has the disadvantage of being resource-intensive [60], especially in terms of human resources, with a teacher/learner ratio of 3/8. The Confidence in Psychiatric Clinical Competence Scale shows acceptable psychometric properties and may be used by other educational teams involved in teaching psychiatry to medical students.

## Supplementary Information

**Additional file 1.**

**Additional file 2.**

**Additional file 3.**

**Additional file 4.**

**Additional file 5.**

## Data Availability

The datasets used and/or analysed during the current study available from the corresponding author on reasonable request.

## Abbreviations

MCQ: Multiple-choice questions
CPCQ Questionnaire: Confidence in Psychiatric Clinical Skills Questionnaire
DASH: Evaluation of debriefing for health simulation
ICC: Intra-class correlation coefficient
ANCOVA: Analyses of covariance
