# Supplementary material for: Evaluating the effectiveness of a single-day simulation-based program in psychiatry for medical students: a controlled study
Source: BMC Med Educ. 2021 Jun 16;21:348. doi: 10.1186/s12909-021-02708-6 (PMC8207590; doi:10.1186/s12909-021-02708-6)
Supplement: Supplementary file 1 — Additional file 1. [file 12909_2021_2708_MOESM1_ESM.docx]

| **Simulation in psychiatry: Scenario 1 « Suicidal attempt »**  *Version 3*  *21/11/2019*  *Authors : Matthieu Roger, Paul Roux* | |
| --- | --- |
| **Learners**   - **Expected level of formation:** medical students, 5^th^ year - **Prerequisite:** having studied at home the chapters “Suicidal risk and behaviours”, “Personality Disorders” and “Alcohol Abuse disorders” of the reference book on psychiatry - **Role**   - 2 residents in psychiatry at the emergency ward   - The remaining 6 students watch the broadcast of the simulated encounter | |
| **Trainers**   - **Who plays the simulated patient:** - **Who runs the briefing:** - **Who runs the pre-briefing:** - **Who plays the facilitators:**    - The senior psychiatrist who can be reached by phone   - The friend Dominique who will intervene in case of blockage, on his own initiative or that of the simulated patient - **Who runs the debriefing:** | |
| **Pedagogical objective** | |
| **Medico-technical:**  - Ask the patient if he/she has suicidal thoughts at a distance from the suicidal attempt  - Search for risk factors for suicide  - Evaluate the degree of suicidal emergency and the dangerousness of the act and propose a treatment adapted to the intensity of the suicidal risk  - Know the diagnostic criteria for borderline personality disorder  - Identify alcohol addiction and propose appropriate treatment | **Interpersonal skills**  - Adapting speech to the patient's reactions (verbal and non-verbal)  - Adopting a reassuring attitude towards a nervous and anxious patient  - Reframing a patient interview that is uninformative due to emotional outbursts  - Propose a therapeutic option compatible with the patient's wishes |
| **Important points to debrief** | |
| The fact that a person has committed a very recent suicidal act should not exempt the clinician from looking for the presence of suicidal ideas that are still active or have disappeared  Does asking questions about the risk of suicide induce suicidal ideas, anxiety and destabilisation in the patient?  "Suicide" is a taboo word, leading to the use of periphrases  How to get out of an impasse: involve the proxy despite the refusal to contact the spouse  The problem of impulsivity and addiction as suicidal risk factors: insufficient grounds for compulsory hospitalisation on their own, but leaving a persistent concern for the family and carers. The value of psychotherapy and addiction treatment in reducing the risk of suicide  Delivering benzodiazepines in emergency departments for alcohol withdrawal: risk of benzodiazepine dependence/misuse? | |
| **References according to the french national program for advanced medical student :**  Item 348: detect situations at risk of suicide in adults  Item 64: diagnose an adjustment disorder, argue the therapeutic attitude and plan the follow-up in adults  Item 64: diagnose a personality disorder, argue the therapeutic attitude and plan the follow-up in adults  Item 74: identify, diagnose and evaluate the repercussions of an alcohol addiction; explain the indications and principles of therapeutic withdrawal; plan the follow-up of the patient | |
| **Briefing :**   - Presentation of the whole optional teaching, its objectives, the need to participate in one of them for each learner. Ask who has already done simulation in psychiatry? - Consent to be signed - Evaluation before: knowledge + self-evaluation of skills - Evaluation after: satisfaction + knowledge + self-evaluation of skills - Reminder of the pedagogical objectives: assessment of suicidal risk, differentiating between depression and acute reaction to a stress factor, personality disorders and addiction - Review of the course of the session with pre-briefing, roleplaying for 10-20 min, debriefing, - Reminder of the rules for the proper functioning of the simulation session:   - Benevolence / mutual respect / constructive criticism   - Confidentiality/anonymity of the evaluations   - Fictional contract and imperfect realism - Presentation of the simulation room, the telephone (number to dial + who can be reached), the possibility of immediately prescribing any treatment by saying it explicitly (name, dosage, galenic), of asking for vitals orally (pulse, blood pressure, T°) - Ask 2 learners to roleplay | |
| **Summary of the situation for trainers**  Suicidal act committed in reaction to a stressor, impulsively without premeditation, facilitated by acute alcohol intoxication. Context of borderline personality traits and alcohol abuse disorder. The trivialisation of the suicidal act by the patient should not lead to an over-reaction on the psychiatrist's side (e.g. compulsory hospitalisation, for instance). | |
| **Pre-Briefing et the context of the situation for learners:**  The doctor of the Short Term Hospitalization Unit of the Virtual Hospital asks you to go and see Camille MARTIN, a 32-year-old patient admitted the day before following a drug auto-intoxication (15 tablets of SERTRALINE and 12 tablets of CYAMEMAZINE) associated with alcohol intoxication. The patient is discharged somatically after 24 hours of monitoring, and you are called for a psychiatric assessment.  Your ER colleague explains that the patient is accompanied by her boyfriend, from whom she is apparently separating. There have been regular bursts of voices coming from her room since her arrival.  He gives you the medical observation and the biological check-up. According to him, there is nothing urgent or worrying at the somatic level. The level of alcohol is 0 according to the breathalyzer. | |
| **Room preparation**  **Environnement:** patient on a stretcher, dressed in a gown, in the simulation room  **Material :**   - medical record with breathanalyser results and biology - a phone to reach the senior psychiatry | |

| **Simulated patient’s description** | | | |
| --- | --- | --- | --- |
| **Last name** | | MARTIN | |
| **First Name** | | Camille | |
| **Sex** | | Female | |
| **Birth / age :** | | 10/11/1987, 32 | |
| **Job** | | Special educator working with children with autism spectrum disorders | |
| **Marital status** (first name and occupation of the spouse) | | In a relationship for one year with Claude, 32 years old, a policeman | |
| **Family situation**  (first names and ages of children) | | A 3-year-old son, Adam, from a previous marriage, in alternating custody | |
| **Social Context** | | You have been living in a 1-bedroom apartment with Claude for 6 months  Your parents have been separated since you were 7 years old, your mother also lives near you. You see her often, although your relationship is conflictual.  Your father lives in the south of France, far from you, you no longer have any contact with him.  You have an older sister aged 40 with whom you do not get on well.  You like sport (fitness, boxing), motorbikes, tattoos and electro music.  A friend Dominique (your tattoo artist) lives near you and can put you up if you need to. | |
| **Medical background - history of illness** | | You have already been to the emergency room several times for similar reasons, pathological drunkenness and scarification.  You have made 3 suicide attempts by voluntary drug intoxication at 17, 22 and 24 years of age.  You have been hospitalised 4 times in a psychiatric clinic for depression, suicidal ideation, suicide attempts and scarifications.  You have already heard of the diagnosis of borderline and bipolar disorders but you don't really know the difference.  Your psychiatrist, Dr Legrand at the mental health centre last saw you a fortnight ago, and your next appointment with him is in two days. At the mental health centre, you also see a psychologist once a week.  You were sexually abused at the age of 13 by a former partner of your mother. You did not talk about it for the first time until you were 19 years old and did not want to report it. Your mother separated from the partner at that time.  Active smoking: 1 pack a day, 10 pack-years  10 years ago, you used cannabis, cocaine, ecstasy, but not at all now  On the other hand, you still drink alcohol regularly. Until now, you have always refused addiction treatment | |
| **Simulation progress** | | | |
|  | **Playing intent - emotional unfolding for the simulated patient** | | **Elements to look for/behave for learners** |
| Basic state | You have been experiencing emotional lability and irritability for several days in the context of conflicts with your partner, who suspects that you have been unfaithful (with your fitness instructor who drives you home after the sessions). Following numerous disputes, also over alcohol consumption, your partner announced last night that he/she intended to leave the house.  You find it difficult to control your emotions and are easily overwhelmed by anger.  You easily answer the psychiatrist's questions, but you digress a lot about your relationship with your partner. You are afraid of being abandoned by your partner and talk about this theme over and over again. | | - Know how to introduce yourself, start the interview in an empathetic way by asking open questions |
| State 1  Suicide risk evaluation | - Exploration of the act: you describe massive anxiety, an intense feeling of emptiness, massive consumption of alcohol, ideas of scarification and suicidal ideas followed by an act in a few minutes in the form of medication ingestion with low suicidal intentionality ("I was ready to do anything to make him/her stay") - Exploration of the current suicidal risk:   - You love your work and your son who is "everything to you". You therefore regret your action. You did not intend to die, even though "it would be easy with the right dose of medication".  - no suicidal family history  - no farewell letter, no change of will  - no more medication at home   - If not assessed, involve Dominic who is concerned about the recurrence of suicide attempt. If the senior psychiatrist is called, he refers the learner to suicide risk assessment | | - Ask precise questions about risk, intentionality and suicidal danger - Know how to frame an interview with a digressive patient |
| State 2  Major depression or adjustement disorder ? | - Outside of the current crisis: no sleep disturbances, no anhedonia, no concentration problems when you are not drunk - Occasional sadness, especially when you feel rejected by others, so not all day. Yesterday, however, you were very sad when you heard about the separation - Low appetite, alcohol suppresses your appetite - Irritable from midday onwards when you think about alcohol | | - know how to differentiate the diagnosis of depression and adjustment disorder |
| State 2 substance abuse disorder | - Start date of consumption: 21 years old - Current quantity consumed: every evening 2 x 12.5 cl glasses of wine and 2 x 25 cl lager + a 50 cl bottle of vodka - From 5pm on weekdays or 10am on weekends (more and more and earlier for several months) - Numerous blackouts, episodes of pathological drunkenness with hetero-aggression and police custody. Large expenses related to alcohol consumption in bars or at home. - Colleagues suspect you have a drinking problem because they saw you shaking on a Monday morning. - If not asked about it by the learners, initiate it by saying that the partner talks about excessive consumption, that you don't necessarily agree, that you don't drink that much, asking what is a reasonable amount to drink... | | - Know how to conduct an interview to evaluate alcohol consumption - Quantify alcohol in standard glasses: 10 g of OH or 10 cl wine, 25 cl beer 4°, 3 cl alcohol at 40° (whisky); formula ° x Vol (L) x 8   - here 25 + 20 + 0.5*30*8 = 45 +120 = 165 g per evening or 1115 g per week, or 112 standard glasses per week  - threshold of standard glasses per week: 21 men and 14 women |
| State 3  Treatment proposition | You are afraid of having to stay in the hospital; you want to be with your son. Trivialisation of the suicidal gesture and the problems linked to alcohol abuse.  You refuse to hear about hospitalisation for alcohol withdrawal.  You agree to call your friend Dominique so that she can drive you home and take you in while you wait to see your psychiatrist in two days. | | - Propose a meeting with the spouse or friend Dominique to find an alternative to hospitalisation - Offer outpatient addiction treatment in addition to the existing follow-up |
| End of the scenario | At what point? When the referral decision is made, as long as the suicidal risk has been explored  In what way? On the initiative of the trainers not playing the patient | |  |
