# Supplementary material for: Evaluating the effectiveness of a single-day simulation-based program in psychiatry for medical students: a controlled study
Source: BMC Med Educ. 2021 Jun 16;21:348. doi: 10.1186/s12909-021-02708-6 (PMC8207590; doi:10.1186/s12909-021-02708-6)
Supplement: Supplementary file 2 — Additional file 2. [file 12909_2021_2708_MOESM2_ESM.docx]

*Supplementary Information 1. Confidence in Psychiatric Clinical skills Questionnaire (CPCQ) .*

| 1. **What is the level of your theoretical knowledge in the field of psychiatry?** | □ Very poor  □ Poor  □ Good  □ Very good |
| --- | --- |
| 1. **How competent do you feel you are in clinical reasoning in psychiatry?** | □ Poorly competent  □ Not very competent  □ Competent  □ Highly competent |
| 1. **How anxious do you feel about conducting a psychiatric interview?** | □ Very anxious  □ Anxious  □ A little anxious  □ Almost not anxious at all |
| 1. **How confident do you feel about your ability to set priorities during a psychiatric interview?** | □ Not confident at all  □ Not very confident  □ Confident  □ Very confident |
| 1. **How competent do you feel about collecting relevant information during a psychiatric interview?** | □ Poorly competent  □ Not very competent  □ Competent  □ Highly competent |
| 1. **How confident do you feel about your ability to share psychiatric information with the patient, his or her entourage, and your colleages?** | □ Not confident at all  □ Not very confident  □ Confident  □ Very confident |
| 1. **How confident are you in your communication skills during a psychiatric interview?** | □ Not confident at all  □ Not very confident  □ Confident  □ Very confident |
| 1. **How well do you feel able to develop and maintain an empathetic attitude during a psychiatric interview?** | □ Poorly capable  □ Not very capable  □ Capable  □ Very capable |
| 1. **How is your knowledge about the different options for managing psychiatric disorders?** | □ Very poor  □ Poor  □ Good  □ Very good |
| 1. **How competent do you feel about translating the national repository knowledge into psychiatric care?** | □ Poorly competent  □ Not very competent  □ Competent  □ Highly competent |
| 1. **How confident do you feel about anticipating problems during psychiatric care?** | □ Not confident at all  □ Not very confident  □ Confident  □ Very confident |
| 1. **How are your skills in measuring the risk-benefit balance in psychiatric care?** | □ Very poor  □ Poor  □ Good  □ Very good |
