# Supplementary material for: Evaluating the effectiveness of a single-day simulation-based program in psychiatry for medical students: a controlled study
Source: BMC Med Educ. 2021 Jun 16;21:348. doi: 10.1186/s12909-021-02708-6 (PMC8207590; doi:10.1186/s12909-021-02708-6)
Supplement: Supplementary file 3 — Additional file 3. [file 12909_2021_2708_MOESM3_ESM.docx]

Supplementary Information 2. Questionnaire on Satisfaction with Simulation-Based Instruction.

| 1. **You would have preferred a lecture instead of a simulation to complete your psychiatric training.** | □ Fully agree  □ Agree  □ Disagree  □ Strongly disagree |
| --- | --- |
| 1. **You would you have preferred role-playing (with some students playing the role of patients) rather than simulation teaching to complement your psychiatric training?** | □ Fully agree  □ Agree  □ Disagree  □ Strongly disagree |
| 1. **How do you situate these sessions compared with the teaching you received in the psychiatric lectures?** | □ Much worse  □ Worse  □ Better  □ Highly superior |
| 1. **How important do you think it is to continue this simulation-based teaching next year?** | □ Very unimportant  □ Unimportant  □ Important  □ Very important |
| 1. **How important do you think it is to make this simulation-based teaching compulsory next year?** | □ Very unimportant  □ Unimportant  □ Important  □ Very important |
| 1. **To what extent were you helped in your learning by actively participating in the simulation?** | □ Helped very litte  □ Helped a little  □ Helped  □ Helped very much |
| 1. **How realistic did you find the scenarios?** | □ Very unrealistic  □ Unrealistic  □ Realistic  □ Very realistic |
| 1. **How much were you helped in your learning by observing the simulation?** | □ Helped very litte  □ Helped a little  □ Helped  □ Helped very much |
| 1. **How much did the debriefing sessions help you in your learning?** | □ Helped very litte  □ Helped a little  □ Helped  □ Helped very much |
| 1. **To what extent did you find the theoretical summary presentations proposed at the end of each session informative?** | □ Very little informative  □ Little informative  □ Informative  □ Highly informative |

For Students who underwent a clerkship in psychiatry:

| **How do you rate these sessions compared to the instruction you received during your clerkship in psychiatry ?** | □ Very low  □ low  □ High  □ Very high |
| --- | --- |
