# Supplementary material for: Evaluating the effectiveness of a single-day simulation-based program in psychiatry for medical students: a controlled study
Source: BMC Med Educ. 2021 Jun 16;21:348. doi: 10.1186/s12909-021-02708-6 (PMC8207590; doi:10.1186/s12909-021-02708-6)
Supplement: Supplementary file 4 — Additional file 4. [file 12909_2021_2708_MOESM4_ESM.docx]

Supplementary Information 3. *Satisfaction with each scenario.*

| 1. **Evaluate the level of difficulty of the first scenario (suicide attempt).**   □ Much too easy  □ Too easy  □ Adapted  □ Too difficult  □ Much too difficult | 1. **Assess the learning value of the first scenario (suicide attempt)?**   □ Very low  □ Low  □ High  □ Very high |
| --- | --- |
| 1. **Evaluate the level of difficulty of the second scenario (bereavement).**   □ Much too easy  □ Too easy  □ Adapted  □ Too difficult  □ Much too difficult | 1. **Assess the learning value of the second scenario (bereavement)?**   □ Very low  □ Low  □ High  □ Very high |
| 1. **Evaluate the level of difficulty of the third scenario (hypomania).**   □ Much too easy  □ Too easy  □ Adapted  □ Too difficult  □ Much too difficult | 1. **Evaluate the learning value of the third scenario (hypomania)?**   □ Very low  □ Low  □ High  □ Very high |
| 1. **Evaluate the level of difficulty of the fourth scenario (school refusal).**   □ Much too easy  □ Too easy  □ Adapted  □ Too difficult  □ Much too difficult | 1. **Evaluate the formative character of the fourth scenario (school refusal)?**   □ Very low  □ Low  □ High  □ Very high |
