# Supplementary material for: Evaluating the effectiveness of a single-day simulation-based program in psychiatry for medical students: a controlled study
Source: BMC Med Educ. 2021 Jun 16;21:348. doi: 10.1186/s12909-021-02708-6 (PMC8207590; doi:10.1186/s12909-021-02708-6)
Supplement: Supplementary file 5 — Additional file 5. [file 12909_2021_2708_MOESM5_ESM.docx]

Supplementary Information 4. Medical Education Research Study Quality Index for the current study.

| 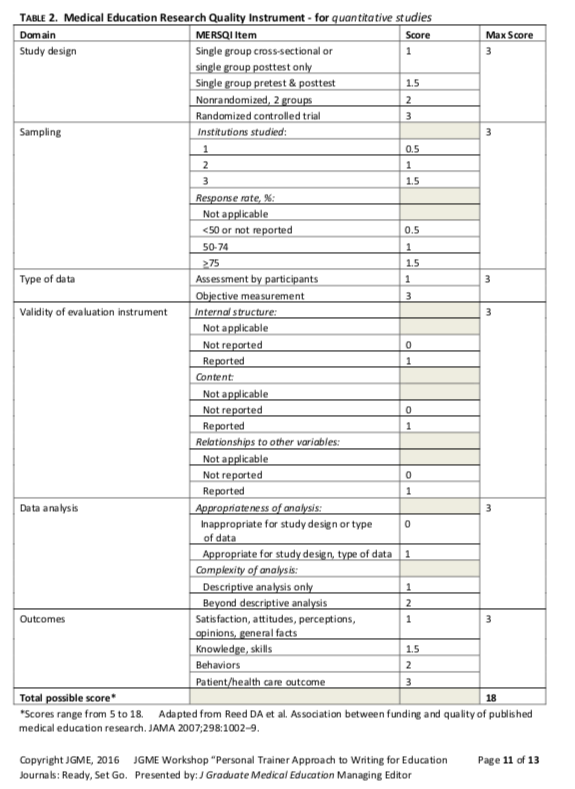 | **Current study**  **2**  **0.5**  **1**  **2**  **1**  **1**  **1**  **1**  **1**  **1.5**  **Total : 12** |
| --- | --- |
